# Raman signatures of *Cnm*-positive *Streptococcus mutans*: III, clinical validation

**DOI:** 10.3389/fmicb.2026.1784129

**Published:** 2026-05-26

**Authors:** Giuseppe Pezzotti, Tetsuya Adachi, Kazunori Kitagawa, Saki Ikegami, Hayata Imamura, Toshiro Yamamoto, Kazu Okuma, Yoshiyuki Matsuo, Wenliang Zhu, Yoshiki Yasukochi, Koichiro Higasa, Saki Nishihama, Hideki Shiba, Miki Kawada-Matsuo, Hitoshi Komatsuzawa

**Affiliations:** 1 Biomedical Engineering Center, Kansai Medical University, Hirakata, Osaka, Japan; 2 International Center for Biomedical Industrial Promotion, Kansai Medical University, Hirakata, Osaka, Japan; 3 Department of Immunology, Graduate School of Medical Science, Kyoto Prefectural University of Medicine, Kamigyo-ku, Kyoto, Japan; 4 Department of Orthopedic Surgery, Tokyo Medical University, Shinjuku-ku, Tokyo, Japan; 5 Department of Molecular Science and Nanosystems, Ca’ Foscari University of Venice, Venice, Italy; 6 Biomarker Disease Laboratory, IRCCS San Camillo Hospital, Via Alberoni, Venice Lido, Italy; 7 Department of Orthopaedic Surgery, Mie University, Graduate School of Medicine, Tsu, Mie, Japan; 8 Department of Dental Medicine, Graduate School of Medical Science, Kyoto Prefectural University of Medicine, Matsugasaki, Kyoto, Japan; 9 Department of Microbiology, Kansai Medical University, School of Medicine, Hirakata, Osaka, Japan; 10 Ceramic Physics Laboratory, Kyoto Institute of Technology, Matsugasaki, Kyoto, Japan; 11 Department of Genome Analysis, Institute of Biomedical Science, Kansai Medical University, Hirakata, Osaka, Japan; 12 Central Research Center, Institute of Biomedical Science, Kansai Medical University, Hirakata, Osaka, Japan; 13 Department of Biological Endodontics, Graduate School of Biomedical & Health Sciences, Hiroshima University, Minami-ku, Hiroshima, Japan; 14 Department of Bacteriology, Graduate School of Biomedical & Health Sciences, Hiroshima University, Minami-ku, Hiroshima, Japan

**Keywords:** clinical isolates, Cnm protein, Raman algorithms, statistical validation, *Streptococcus mutans*

## Abstract

This study sought to confirm the possibility of using Raman spectroscopy for oral health management by investigating molecular fingerprints of *Streptococcus mutans* bacteria obtained from clinical samples. A randomized, double blind study was conducted on 30 samples obtained from clinical subjects (*n* = 30) in search for Cnm-positive strains, while concurrently and independently performing genomic analyses on the same samples. Samples were characterized after biofilm purification and following a 24 h cycle of *in vitro* stabilization in absence or in presence of sucrose. Following the basic spectroscopic assessments described in two companion papers, the molecular structures of the isolates were analyzed according to quantitative spectroscopic parameters related to the presence of oxysulfur molecules, the degree of disorder of protein secondary structure, and the level of simplification of *N*-acetylglucosamine in membrane structure in favor of disordered proteins, based on a previously established spectroscopic assessment framework. Spectroscopic and genomic approaches converged in locating (the same) 4 Cnm-positive isolates over a total sampling of 30 (~13%). Clinical validations confirmed that Cnm-positive strains all had higher protein disorder, higher degree of sulfoxidation, and higher levels of peptidoglycan structural simplification as compared to the Cnm-negative ones. These structural characteristics contribute to an increased level of virulence in the isolate, regardless of its bacterial serotype. Besides matching the results of Cnm genomic analyses, Raman assessments added important information on the pathophysiological stress state of the bacterial isolate and opened the way to fast and insightful diagnostics of the oral flora. The present investigation clearly demonstrated the advantages of Raman technology as compared to traditional culture/molecular detection, in terms of speed, label-free, state sensitivity, and application to specific scenarios, such as high-risk caries risk screening and rapid strain virulence typing.

## 1 Introduction

Modern preventive medicine in oral care is not just about keeping teeth and gums healthy; it also involves safeguarding overall health, reducing healthcare costs, and improving quality of life (Sischo and Broder, 2011). Nowadays, it is becoming increasingly clear how, through regular oral hygiene practices, professional care, and lifestyle adjustments, not only the risk of developing oral and systemic health problems could be reduced, but also healthy longevity standards could significantly be improved. Being oral health intimately connected to overall health, oral flora and untreated dental diseases link to various systemic conditions, including heart disease, ischemic strokes, respiratory infections, and dementia (Peng et al., 2022). Oral microbiome is thus crucial to health and its monitoring is key in developing targeted therapies, which will ultimately support the development of a personalized and preventive medicine approach (Nimish Deo and Deshmukh, 2019). However, preventing diseases before they occur (rather than treating them after they develop) requires substantial technological progress, most of which is nowadays yet in its embryonal stage.

Raman spectroscopy holds significant potential in the field of preventive oral healthcare (Zhang et al., 2022). This method leverages the scattering of light to provide detailed information about the molecular composition and structure of biological materials. The Raman approach could be extremely useful for an early detection of oral health problems that may otherwise go unnoticed until the damages they cause reaches an irreparable stage. By enabling early intervention and personalized care, Raman spectroscopy could play a key role in improving patient outcomes, reducing healthcare costs, and ultimately promoting better long-term health. The advantage of using Raman spectroscopy in oral flora diagnostics lies on the rapid and non-invasive way that this technique provides in analyzing the biochemical composition of oral microorganisms (Pezzotti, 2021; Pezzotti et al., 2023a; Rebrosova et al., 2022). Upon detecting vibrational fingerprints of key molecules belonging to the structure of pathogens, Raman spectroscopy allows identifying in nearly real time different bacterial and fungal species (Ho et al., 2019; Pezzotti et al., 2022a, 2022b), assessing their metabolic states (Pezzotti et al., 2022a, 2022b, 2023b, 2024), and analyzing the structure and toxicity of their biofilms and exosomes (Pezzotti et al., 2023a, 2023b). The Raman approach can be particularly useful in detecting pathogenic bacteria or monitoring changes in oral microbiome associated with various conditions, such as periodontal disease or oral infections. For example, Raman spectroscopy could clarify the composition and structure of biofilms formed by oral bacteria both in isolated and competitive conditions between *Streptococcus mutans* and *Streptococcus sanguinis* (Pezzotti et al., 2023b). This helps understanding how biofilms contribute to diseases and can in turn inform treatment strategies.

In two companion papers (Pezzotti et al., 2026a, b), we detailed a Raman characterization of *S. mutans*, the oral pathogen commonly associated with dental caries (Hamada and Slade, 1980; Loesche, 1986). In taking one step forward, we succeeded in locating several spectroscopic parameters that can allow differentiating its Cnm-positive (*Cnm*^(+)^*Sm*) from Cnm-negative (*Cnm*^(−)^*Sm*) strains. This accomplishment exploited the vibrational fingerprints of a structural mutation that enables the bacterium to enhance its adhesion capability to soft tissue. The Cnm protein is associated with virulence traits, and differentiates the biochemical composition and metabolic profiles of the bacterial strain. *Cnm*^(+)^*Sm* bacteria, besides inducing more aggressive forms of tooth decay as compared to the *Cnm*^(−)^*Sm* ones, could enter the bloodstream and cause intracerebral hemorrhage and deep microbleeds, as originated from their superior ability to adhere to the tunica intima in the innermost layer of the veins (Abranches et al., 2006; Nakano et al., 2011; Tonomura et al., 2016; Aviles-Reyes et al., 2017; Otsugu et al., 2017; Hosoki et al., 2020; Naka et al., 2022; Taniguchi et al., 2022). By analyzing the Raman signatures of clinical isolates, we succeeded in identifying the unique molecular vibrations associated with specific cellular components and/or metabolic byproducts that set apart *Cnm*^(+)^*Sm* strains. This spectroscopic procedure provided us with a rapid and non-invasive method for distinguishing between the two bacterial types, namely, a key information in understanding their different roles in dental caries and, concurrently, in assessing the risks for the circulatory diseases they might induce.

In this paper, we apply the Raman algorithms developed in the two companion papers and describe in details the outputs of a large-scale, double blind genomic/spectroscopic analysis performed on a statistically significant number of clinical isolates. The isolates included in the present results belonged to different bacterial serotypes, which enabled testing the generality of the proposed Raman multiomic approach to bacterial differentiation. The present data could be seen as a final confirmation for the suitability of the proposed Raman procedure in efficiently assessing the intrinsic virulence of *S. mutans* clinical isolates. Based on this clinical confirmation, this study opens the way to a new important application in future oral diagnostics with the Raman probe.

## 2 Experimental procedures

### 2.1 Clinical isolates and their double-blind testing procedure

*Streptococcus mutans* clinical isolates (KSM strains) were obtained as explained in a previous study (Watanabe et al., 2021). *Streptococcus mutans* (*S. mutans*) strains were isolated from saliva collected from volunteers. Isolation of *S. mutans* was approved by the ethics committee of the Kagoshima University (No. 701) and ethical committee for Epidemiology of Hiroshima University (E-1998). Written informed consent was obtained from all participants. All methods were performed in accordance with the approved guidelines and regulations.

A group of 30 randomly selected volunteer patients was analyzed without *a priori* knowing the type of bacteria they held in their swabs. These same swab samples were then shared between two independent research groups: Hiroshima University for genomic analysis and Kansai Medical University for Raman spectroscopic analysis. The procedure was double blinded, so that the two research groups independently made the respective analyses without communicating to each other. Then, when the analytic procedures ended, the results were sealed, and then shared only at the end of all procedures.

### 2.2 Whole genome sequence analysis

*Streptococcus mutans* chromosomal DNA was extracted according to a previously developed method (Shibata et al., 2003) and analyzed according to a previously published procedure (Kawada-Matsuo et al., 2013), as already described in the companion Part I and Part II (Pezzotti et al., 2026a, b).

Serotype, virulence genes (*cnm, gtfb, gtfC, gtfD* and *Pac*) and Mutacin genes (Mutacin I, II, III, IIIb, IV, K8 and SmB) were identified using whole genome sequence (WGS) of *S. mutans* strains (Watanabe et al., 2021). Each serotype was determined according to a previously developed method (Shibata et al., 2003; Nakano et al., 2004). *Cnm* (Accession No. AB102689.1), *gtfB-D* (Accession No. NC_004350.2) and *Pac* (Accession No. AB548069.1) genes were searched as reference genes. Each gene was identified using the software ABRicate v.1.0.1.^3^ Mutacin genes were searched for based on the nucleotide sequences described elsewhere (Watanabe et al., 2021). More details are reported in the companion Part II (Pezzotti et al., 2026b).

### 2.3 Biofilm assay

In order to investigate biofilm formation, a crystal violet staining assay was used. An aliquot of 100 μL of TSB supplemented with 2% sucrose was added to the wells of a 96-well flat bottom microplate (Greiner Bio-One, Germany). Overnight cultures of *S. mutans* strains were adjusted to 1.0 (10^9^ cells/mL) at Optical Density at 660 nm. After 100-fold dilution with fresh TSB, 10 μL (10^5^ cells) were inoculated into each well, and the plate was incubated at 37 °C for 24 h. Bacterial medium in each well was removed and the biofilms were rinsed with phosphate-buffered saline (PBS) three times. Then, 100 μL of 0.1% crystal violet solution was added to each well and left for 10 min. Then, the crystal violet solution was removed followed by rinsing in PBS three times to remove excess dye. After air-drying, the stained biofilms were solubilized in 100 μL of 33% acetic acid for 15 min. Finally, the optical density (OD) was determined at OD_570_ nm with an iMark Microplate reader (Bio-Rad Laboratories, Hercules, CA, USA).

### 2.4 Raman spectroscopy

Raman spectra were collected *in situ* on living *S. mutans* bacterial samples collected from swab samples of 30 different patients. The samples were isolated and cleaned according to the procedures described above. All sample preparation and Raman characterization procedures were exactly the same as that described in the two companion papers (Pezzotti et al., 2026a, b). Briefly, bacterial samples were analyzed after 2 days culture both in absence and in presence of sucrose. Raman data were collected by means of a high spectrally resolved spectrometer designed for measurements on biological samples (LabRAM HR800, Horiba/Jobin-Yvon, Kyoto, Japan). The equipment was set in confocal mode with a 20× objective lens and the wavelength of the incoming light was 532 nm, as generated by a solid-state laser source operating at 10 mW. We found no observable spectral changes in our measurements with a 10 mW green laser over 10 s in comparison with both shorter and longer time exposures, confirming that these laser irradiation conditions are well below typical photodamage thresholds, in agreement with what reported in literature for visible Raman excitation of bacteria (Yuan et al., 2018).

The Raman scattered light was monitored by means of a single monochromator interfaced with an air-cooled charge-coupled device (CCD) detector (Andor DV420-OE322; 1,024 × 256 pixels). The acquisition time for a single spectrum was typically 10 s for three successive acquisitions at each location. A spectral resolution of better than 1 cm^−1^ was achieved by concurrently collecting (at each measurement) an internal reference signal from a selected neon lamp to calibrate the spectrometer. Series of 10 spectra were systematically collected at different locations (over a total area of ~2 mm^2^) on each bacterial sample and averaged in order to obtain a statistically representative spectra for each bacterial isolate. Experimental Raman spectra were subjected to polynomial baseline subtraction and deconvolution into series of Gaussian-Lorentzian sub-band components. The baseline subtraction procedure was performed using options available in commercial software (LabSpec 4.02, Horiba/Jobin-Yvon, Kyoto, Japan) with fixed criteria for all collected spectra. Spectral deconvolution criteria were based on a self-developed machine-learning algorithm, which has been reported in previously published papers (Pezzotti, 2021; Pezzotti et al., 2021).

### 2.5 Statistical analyses

The statistical relevance of the parameters extracted from the Raman experiments was analyzed by computing mean values and standard deviations. After confirming homogeneity of variances among groups, the statistical validity was evaluated by applying the unpaired Student’s *t*-test values *p* < 10^−2^ and *p* < 10^−3^ were considered as statistically significant and labeled with two and three asterisks, respectively.

## 3 Experimental results

### 3.1 Identification of virulence genes and biofilm formation

Table 1 lists the 30 bacterial isolates analyzed in this study. Serotypes, *Cnm*, and other virulence genes were identified upon screening the whole genome sequence *s*. Among 30 strains, the number of strains with serotype *c*, *e*, *k* and *f* were 25, 6, 3 and 1, respectively. More in details, 5 strains showed double positive serotype including *c* + *e* (2 strains), *c* + *k* (2 strains) and *c* + *f* (1 strain); only 4 strains were positive for *cnm* gene, whose product is a collagen binding protein (Okahashi et al., 1989), while *gtfB-D* and *PAc* genes were all positive. Regarding Mutacin genes, 21 and 6 strains resulted single and double positive, respectively, while 3 strains did not have these genes. Among the investigated 30 strains, Mutacin IV (16 strains), Mutacin Smb (5 strains), Mutacin I (4 strains), Mutacin K8 (4 strains), Mutacin IIIb (2 strains) and Mutacin II (2 strains) were identified.

**Table 1:** Genomic characteristics of the 30 *S. mutans* clinical isolates analyzed in this study; serotypes, *Cnm* and other virulence genes, and mutacin genes were identified upon screening the whole genome sequence *s* (labels + and – indicate presence and absence of each specific gene, respectively; PMID origin also specified).

Sample	Sample label	Sero type	Biofilm assay	Virulence gene					Mutacin	Origin
				*Cnm*	*gtfB*	*gtfC*	*gtfD*	*PAc*		
1	KSM34	c	0.171	−	+	+	+	+	I	34155274
2	KSM114	c	0.302	−	+	+	+	+	IIIb, IV	34155274
3	KSM189	c	0.470	−	+	+	+	+	IV	34155274
4	KSM153	c,e	0.479	+	+	+	+	+	IV, K8	34155274
5	KSM43	c	0.547	−	+	+	+	+	IV	34155274
6	KSM110	c,k	1.141	−	+	+	+	+	K8	34155274
7	KSM188	c	1.142	−	+	+	+	+	Smb	34155274
8	KSM170	c	1.167	−	+	+	+	+	IIIb	34155274
9	KSM55	c	1.207	−	+	+	+	+	K8	34155274
10	KSM112	e	1.211	−	+	+	+	+	IV, Smb	34155274
11	KSM146	c,k	1.555	−	+	+	+	+	−	34155274
12	KSM101	c	1.599	+	+	+	+	+	I	34155274
13	KSM80	c	1.606	−	+	+	+	+	K8	34155274
14	KSM120	c	1.613	−	+	+	+	+	IV	34155274
15	KSM172	c	1.669	−	+	+	+	+	IV, Smb	34155274
16	KSM5	k	1.685	+	+	+	+	+	IV	34155274
17	KSM13	c	1.687	−	+	+	+	+	IV	34155274
18	KSM58	c	1.704	−	+	+	+	+	IV	34155274
19	KSM51	c	1.711	−	+	+	+	+	II	34155274
20	KSM92	c,f	1.759	−	+	+	+	+	−	34155274
21	KSM175	e	2.298	−	+	+	+	+	IV, Smb	34155274
22	KSM137	c	2.344	−	+	+	+	+	IV	34155274
23	KSM159	c	2.383	−	+	+	+	+	IV	34155274
24	KSM187	c	2.388	−	+	+	+	+	Smb	34155274
25	KSM62	e	2.430	−	+	+	+	+	IV	34155274
26	KSM229	c	3.439	+	+	+	+	+	I	34155274
27	KSM207	c	3.457	−	+	+	+	+	I, II	34155274
28	KSM201	c,e	3.499	−	+	+	+	+	−	34155274
29	KSM200	e	3.500	−	+	+	+	+	IV	34155274
30	KSM238	c	3.500	−	+	+	+	+	IV	34155274

The results of biofilm assay are also shown as determined according to OD570 nm analyses on samples cultured with sucrose addition. Sample labels correspond to those used in all figures.

Unlike clinical strains cultured without sucrose, strains cultured with addition of sucrose made us available samples of biofilm, which were analyzed by a standard biofilm assay (cf. Section 2.2). The biomass of *S. mutans* biofilm varied significantly among isolates, showing wide variations from low to high biofilm formation. The values recorded at OD_570_ ranged from 0.171 to 3.50. The mean value among 30 strains was 1.832 ± 0.994. Since biofilm formation activity did not show any correlation with serotypes and *cnm* gene, they were not included in Table 1. The full data set and further discussion on biofilm data will be offered in the context of a comparison with biofilm Raman data in the forthcoming Section 3.3.

### 3.2 Raman spectra of clinical isolates cultured without or with sucrose

#### 3.2.1 Clinical isolates cultured without addition of sucrose

**Figure 1:**
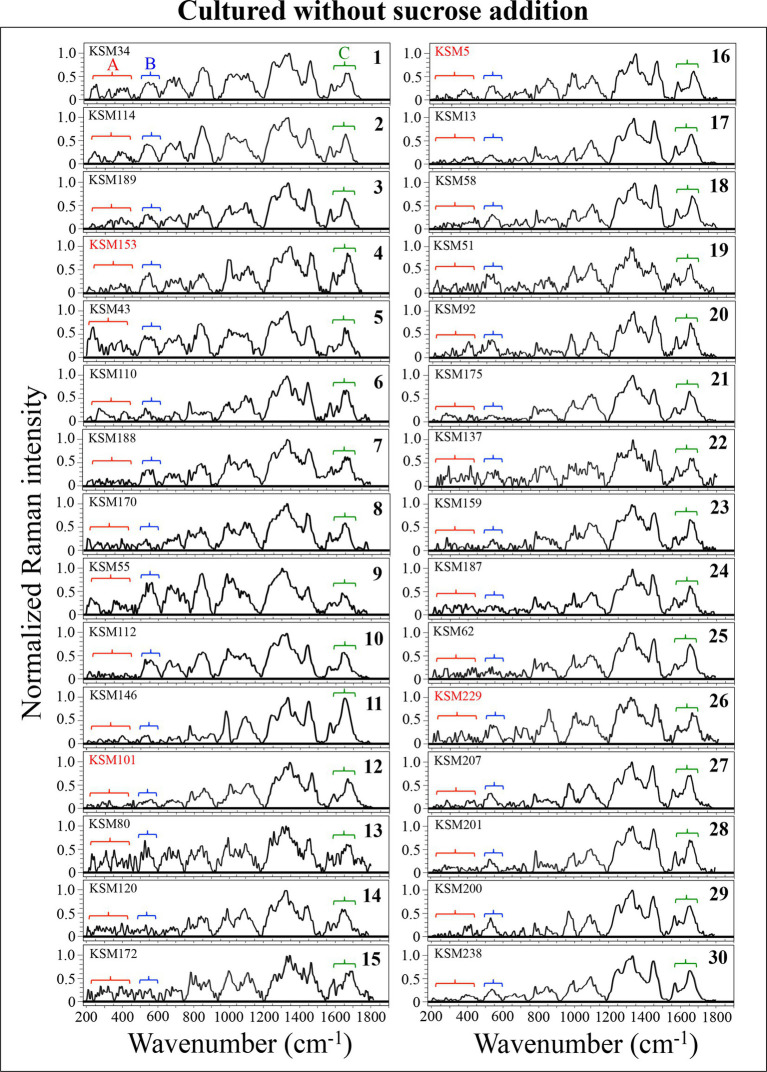
Average Raman spectra in the wavenumber interval 200–1,800 cm^−1^ collected on 30 clinical isolates from swab samples of different patients after biofilm cleaning and successive culture for 24 h without sucrose addition (cf. labels in inset corresponding to those assigned in Table 1); each spectrum is the average of 10 spectra collected at different locations on the same sample (total area of ~2 mm^2^). Three main wavenumber sub-zones are labeled in inset as A (225,450 cm^−1^), B (480,620 cm^−1^), and C (16,301,700 cm^−1^), their spectral variations revealing key differences in molecular structure among clinical isolates regarding oxysulfur anions, *GlcN* and *GlcNAc*, and protein secondary structures, respectively. Red labels locate the 4 clinical strains classified as *Cnm*^(+)^*Sm* according to genomic analyses (i.e., samples labeled as 4, 12, 16, and 26 in Table 1).

In Figure 1, average spectra are shown as measured in the wavenumber interval 200–1,800 cm^−1^ for each of 30 clinical isolates from different patients (cf. labels in inset corresponding to those assigned in Table 1). The presented spectra, each being the average of 10 at different locations on the same sample (total area of ~2 mm^2^), were collected on samples subjected to biofilm cleaning and successive culture for 24 h without addition of sucrose. Since *S. mutans* can only exploit the extracellular enzyme glucosyltransferase B in order to produce biofilm glucans from sucrose (Lemos et al., 2019), in absence of sucrose in the culture, none of the examined clinical isolates was able to produce additional biofilm. Accordingly, it can be assumed that the collected Raman signals mainly belong to the intrinsic structure of the bacterial cells. Spectra from all clinical isolates, as shown in Figure 1, displayed high morphological similarity, but clearly differed in a number of fundamental details. According to findings shown in two companion papers (Pezzotti et al., 2026a, b), three main wavenumber sub-zones (i.e., labeled in inset as A, B, and C, respectively) were attentioned. They correspond to the spectral intervals 225–450, 480–620, and 1,630–1,700 cm^−1^, respectively. Spectral variations in the above zones can reveal key differences in molecular structure among clinical isolates, specifically regarding oxysulfur anions, glucosamine (*GlcN*) and *N*-acetylglucosamine (*GlcNAc*) molecules (in the peptidoglycan external membrane), and protein secondary structures, respectively. Zone A specifically contains S–S stretching and other sulfur-related signals arising from a variety of different oxysulfur compounds (Sato et al., 1985), Zone B includes several skeletal-mode signals from *GlcN* and *GlcNAc* molecules (She et al., 1974), while Zone C is representative of the Amide I mode, which characterizes the secondary structure, and thus the degree of disorder, of the overall bacterial proteins (Krimm and Bandekar, 1986; Dong et al., 1990; Rygula et al., 2013; Sadat and Joye, 2020). Differences in the above three zones are key in estimating the virulence of the clinical isolates and reflect their different pathological impact on human health. Specific differences among clinical isolates will be analyzed in the next section and discussed in Section 4.1, in order to screen the possibility of linking structural differences to genomic categories.

#### 3.2.2 Clinical isolates cultured with addition of sucrose

**Figure 2:**
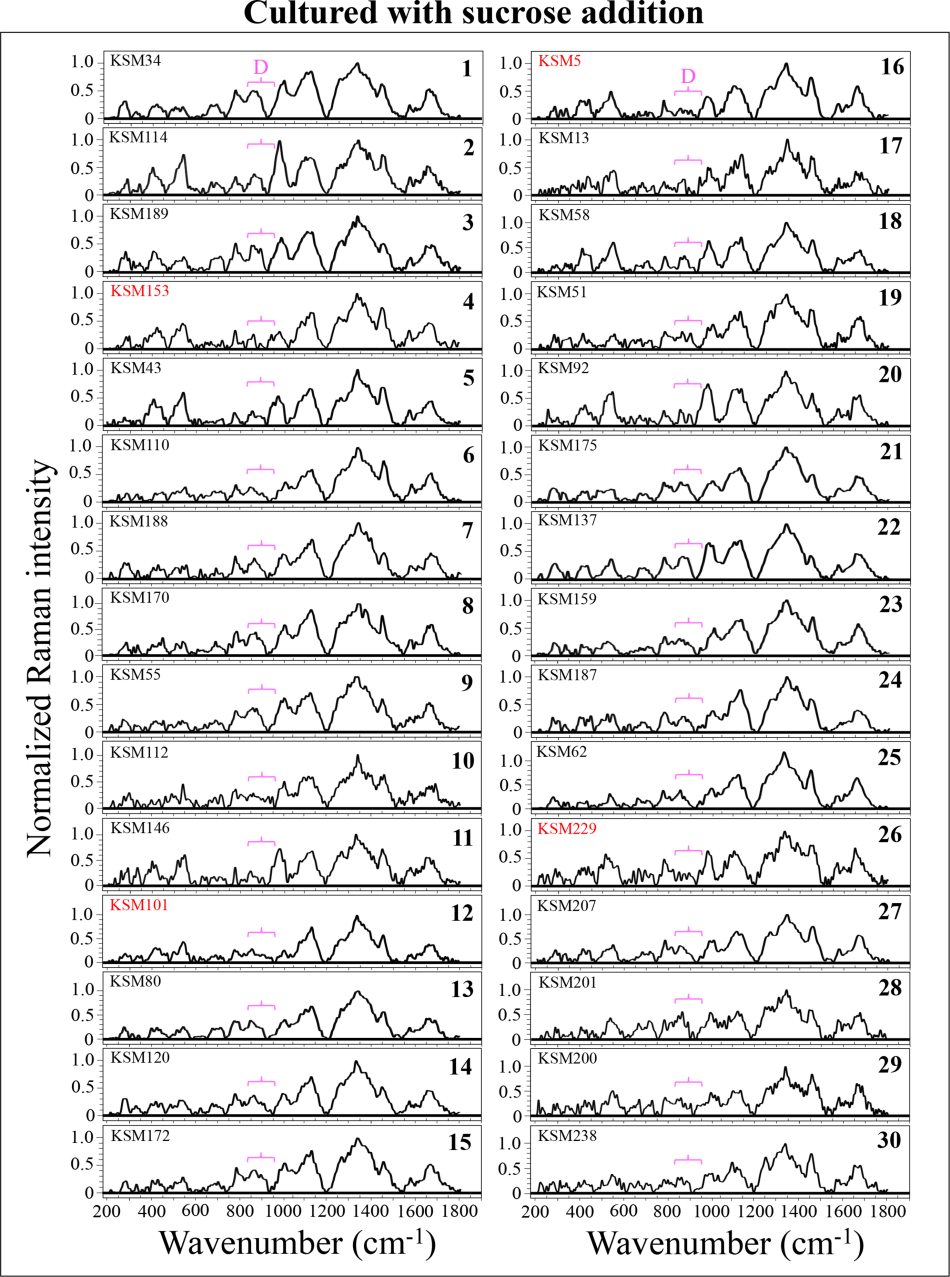
Average Raman spectra in the wavenumber interval 200–1,800 cm^−1^ collected on 30 clinical isolates from swab samples of different patients after biofilm cleaning and successive culture for 24 h with addition of sucrose (cf. labels in inset corresponding to those assigned in Table 1); each spectrum is the average of 10 spectra collected at different locations on the same sample (total area of ~2 mm^2^). The spectral interval 850–950 cm^−1^ (cf. labeled as D in inset) contains key signals characterizing the structure of biofilm glucans and a fingerprint signal at ~851 cm^−1^ reflecting the presence of α-glucose rings from free sugar molecules as a marker of the available reserve energy stored within the biofilm. Red labels locate the 4 clinical strains classified as *Cnm*(+)*Sm* according to genomic analyses (i.e., samples labeled as 4, 12, 16, and 26 in Table 1).

Figure 2 shows the Raman spectra collected in the spectral region 200–1,800 cm^−1^ for each of 30 clinical isolates from different patients (cf. labels in inset corresponding to those in Table 1). Each presented spectra represents the average of 10 spectra collected at different locations on the same isolate sample (total area of ~2 mm^2^). In this case, the clinical isolates were cultured for 2 days with addition of sucrose. When isolates are cultured with adding sucrose, the Raman spectrum becomes dominated by signals arising from the biofilms copiously produced during culture through bacterial glucosyltransferase B activity. Similar to the case of Figure 1, the spectra from different clinical isolates in Figure 2 were all morphologically similar, but clearly differed with respect to the relative intensities of their common signals. Following our previous analyses (Pezzotti et al., 2026a, b), we attentioned Zone D in the spectral interval 850–950 cm^−1^ (cf. labels in inset), which contains key signals characterizing the structure of glucans. More specifically, signals at ~890, 919, and 941 cm^−1^ represent markers for β-glucans, α-1,6-and α-1,3-linked glucans, respectively (Wiercigroch et al., 2017). An additional signal located at ~851 cm^−1^ in the same zone reflects the presence of α-glucose rings from free sugar molecules, which thus represents the reserve of available energy stored within the biofilm. Linking spectral signals to different fractions of glucans in the biofilm provides important hints on the survival “strategy” that each specific strain adopts, in terms of biofilm protective stiffness and impermeability toward drugs and degrading enzymes. The biofilm structure is expected to be influenced by environmental conditions and thus to reflect the pathophysiological state of the isolate. Specific spectral differences in the spectral Zone D will be analyzed in details in the next section and further discussed in the forthcoming Section 4.1.

### 3.3 Raman spectral analyses in specific wavenumber intervals

Following exactly the same procedure shown in the companion Part II paper (Pezzotti et al., 2026b), we further collected Raman spectra with high spectral resolution in zones A–C on the 30 clinical isolates under examination.

#### 3.3.1 Clinical isolates cultured without addition of sucrose

**Figure 3:**
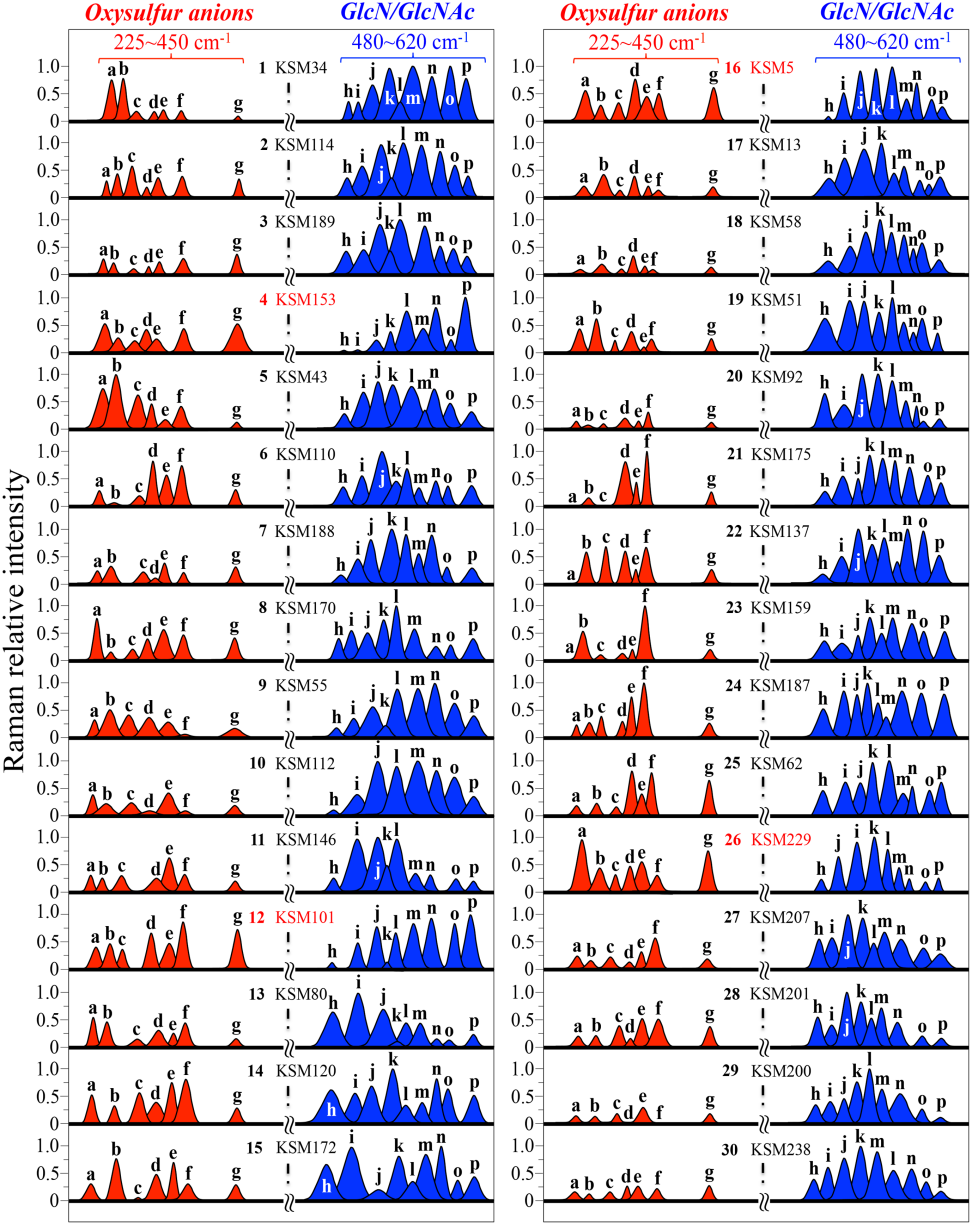
Oxysulfur spectral signals from Zone A (225–450 cm^−1^; left side) and *GlcN*/*GlcNAc* molecules from Zone B (480–620 cm^−1^; right side), as extracted from high spectrally resolved spectra (average of 10 at different locations) of 30 bacterial isolates from swab samples after biofilm cleaning and successive culture for 24 h without addition of sucrose (cf. labels in inset corresponding to those assigned in Table 1); 7 signals from S-related bonds in oxysulfur molecules, labeled as a (230 cm^−1^), b (250 cm^−1^), c (270 cm^−1^), d (288 cm^−1^), e (320 cm^−1^), f (330 cm^−1^), and g (450 cm^−1^) are examined for Zone A (cf. vibrational origins in Section 3.3) and 9 skeletal-mode signals at 487, 511, 526, 545, 563, 569, 581, 597, and 605 cm^−1^ (from C–O–C/C–C–C bending and ring deformation) in *GlcN*/*GlcNAc* molecules, labeled as h–p, respectively, are attentioned for Zone B. Signals from Zones A and B were normalized with respect to their respective maxima and plotted with maintaining their relative intensity. Red labels locate the 4 clinical strains classified as *Cnm*^(+)^*Sm* according to genomic analyses (i.e., samples labeled as 4, 12, 16, and 26 in Table 1).

Average and deconvoluted spectra are shown in Figure 3 for all clinical samples (cf. labels in inset) in spectral Zones A and B. Following the outputs of two companion papers (Pezzotti et al., 2026a, b), we focused on signals from oxysulfur molecules, *GlcN* and *GlcNAc* molecules, and Amide I protein secondary structures.

Oxysulfur spectral signals extracted from Zone A (left side) belonged to: tetrathionate ions (S_4_O_6_^2−^) with three possible S–S bond stretching (S1–S2, S2–S3, and S3–S4) at around 230, 270, and 310 cm^−1^ (labeled as a, c, and e in inset to Figure 3), disulfite ions (S_2_O_5_^2−^) with a single S–S stretching signal at ~270 cm^−1^ (labeled b and fully overlapping the S_4_O_6_^2−^ signal), dithionate ions (S_2_O_6_^2−^) with S–S stretching at 288 cm^−1^ (labeled d) and SO_3_ rocking at 320 cm^−1^ (labeled e and partly overlapping S3–S4 stretching in S_4_O_6_^2−^), dithionite ions (S_2_O_4_^2−^) with S–S stretching at 250 cm^−1^ (labeled b) and SO_2_ twisting at 330 cm^−1^ (labeled f), and thiosulfate ions (S_2_O_3_^2−^) with S–S stretching at 450 cm^−1^ (labeled g) (Sato et al., 1985).

On the right side of Figure 3, we report the 9 skeletal-mode signals (i.e., C–O–C/C–C–C bending and ring deformation modes) belonging to the *GlcN* and *GlcNAc* molecules contained in the peptidoglycan structure of the bacterial membrane (She et al., 1974; Wiercigroch et al., 2017), as retrieved from Zone B of each isolate spectrum (labeled as h–p in inset to Figure 3).

**Figure 4:**
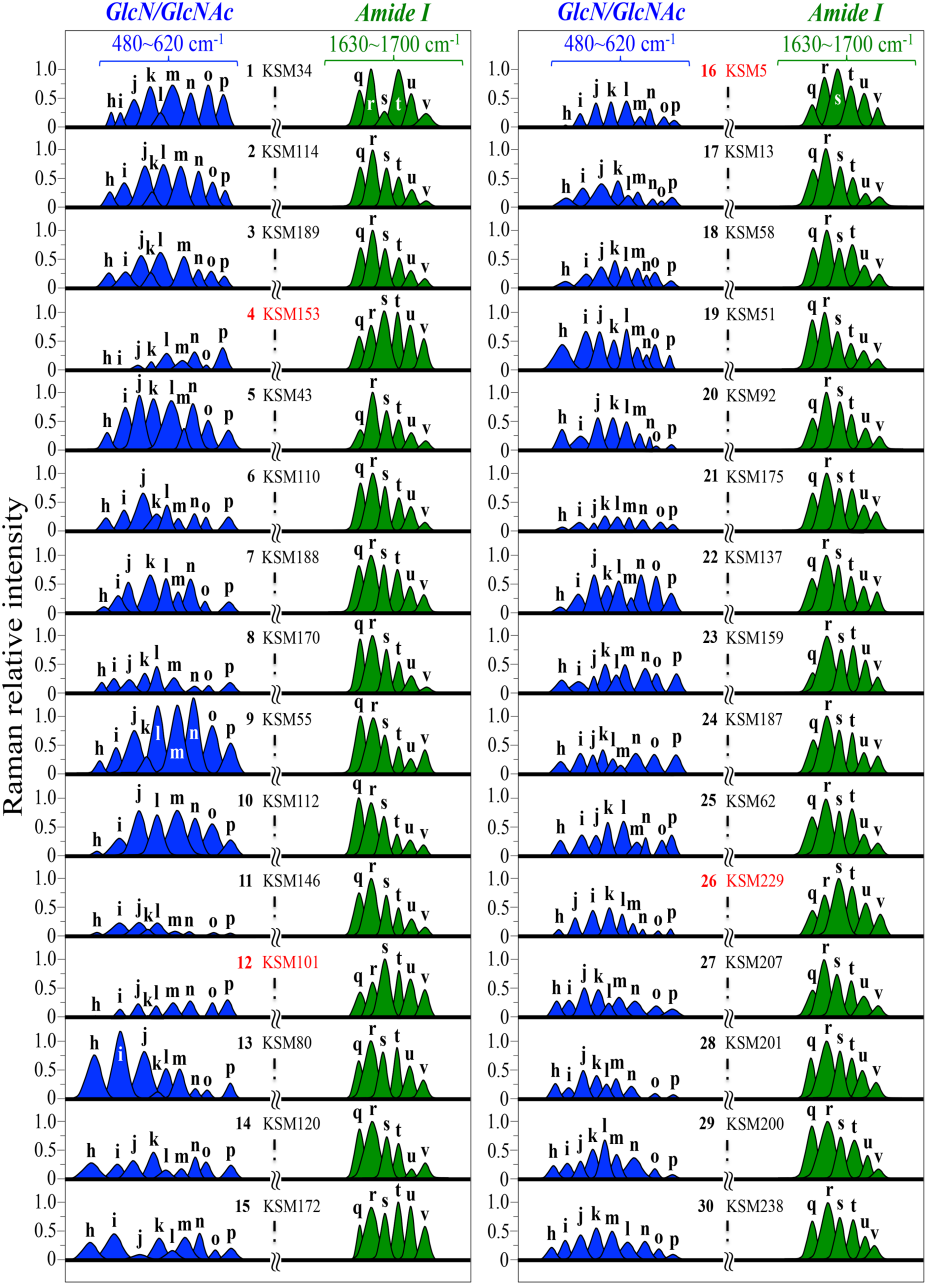
With the same procedure adopted for Figure 3, *GlcN*/*GlcNAc* signals from Zone B (i.e., the same as those given in Figure 3) are extracted and compared with Amide I signals in Zone C (1,630–1,700 cm^−1^) representing the secondary structures of bacterial proteins (bands labeled r from α-helix at ~1,649 cm^−1^; q and t from β-sheet at ~1,636 and ~1,671 cm^−1^, respectively; s from random coil at ~1,660 cm^−1^; and, u and v from β-turn at ~1,684 and ~1,695 cm^−1^, respectively). Signals from Zones B and C were normalized with respect to their respective maxima and plotted with maintaining their relative intensity. Red labels locate the 4 clinical strains classified as *Cnm*^(+)^*Sm* according to genomic analyses (i.e., samples labeled as 4, 12, 16, and 26 in Table 1).

With a similar procedure, Amide I signals comprised were extracted from high spectrally resolved (average) spectra collected in Zone C for each isolate sample. Figure 4 shows such signals in comparison with *GlcN*/*GlcNAc* signals from the respective samples (i.e., the same as those given in Figure 3). The Amide I signals represents the secondary structures of the bacterial proteins, as follows: α-helix (at ~1,649 cm^−1^; labeled r in inset to Figure 4), β-sheet (at ~1,636 and ~1,671 cm^−1^; labeled q and t, respectively), random coil (at ~1,660 cm^−1^; labeled s), and β-turn (~1,684 and ~1,695 cm^−1^; labeled u and v, respectively) (Krimm and Bandekar, 1986; Dong et al., 1990; Rygula et al., 2013; Sadat and Joye, 2020).

**Figure 5:**
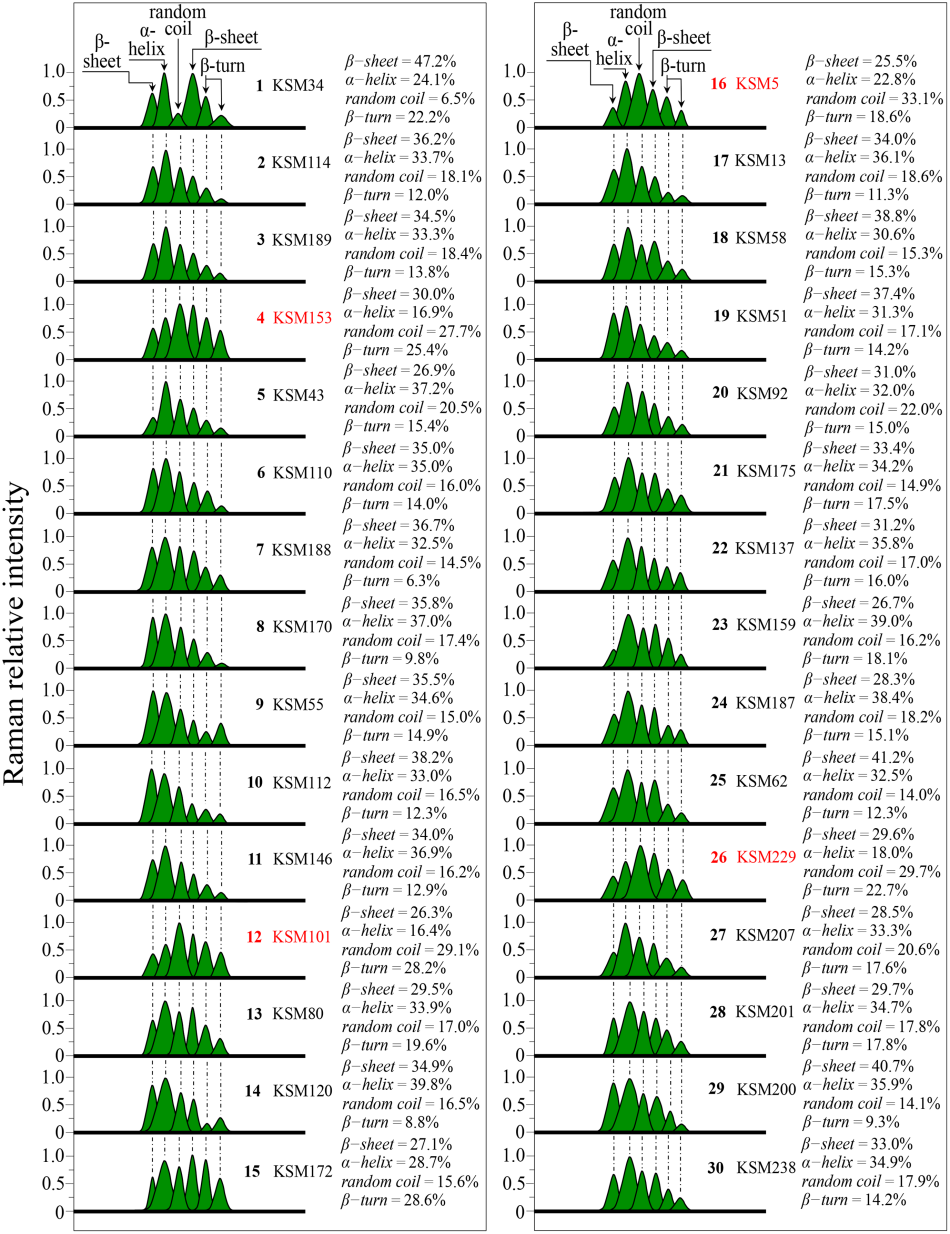
Computation of the fractions of different secondary protein structures (cf. percent values in inset) for the 30 bacterial samples under examination (the same as those shown in Figure 4; cf. labels in inset), as computed from the relative areal fraction of Raman bands belonging to each secondary structure with respect to the total amide I area. Red labels locate the 4 clinical strains classified as *Cnm*^(+)^*Sm* according to genomic analyses (i.e., samples labeled as 4, 12, 16, and 26 in Table 1).

Signals from Zones A and B (in Figure 3) and Zones B and C (in Figure 4) were normalized with respect to their respective maxima and plotted with maintaining their relative intensity. Figure 5 gives the fractions of different secondary protein structures (cf. percent values listed in inset) for different bacterial isolate samples (cf. labels in inset), as computed from the relative areal fractions of each secondary structure with respect to the total Amide I area. From comparing signals from different isolate samples in Figures 3–5, it appears immediately clear that not only the relative intensities among bands within the respective Zones A, B, and C differ, but also that the overall spectral balances oxysulfur/peptidoglycan signals and peptidoglycan/Amide I signals could significantly swing among different samples. This latter circumstance points to a significant fluctuation in the bacterial structure, as discussed in the next section. In particular, the 4 clinical strains classified as *Cnm*^(+)^*Sm* according to genomic analyses, namely, the samples labeled as 4, 12, 16, and 26 in Table 1, experienced clearly stronger oxysulfur signals (cf. Figure 3) and a predominance in random coil secondary protein structure (i.e., higher structural disorder; cf. Figure 5).

#### 3.3.2 Clinical isolates cultured with addition of sucrose

In order to check possible differences in biofilm structure, similar Raman characterizations were applied to the 30 clinical isolate samples after culturing them for 24 h with sucrose addition.

**Figure 6:**
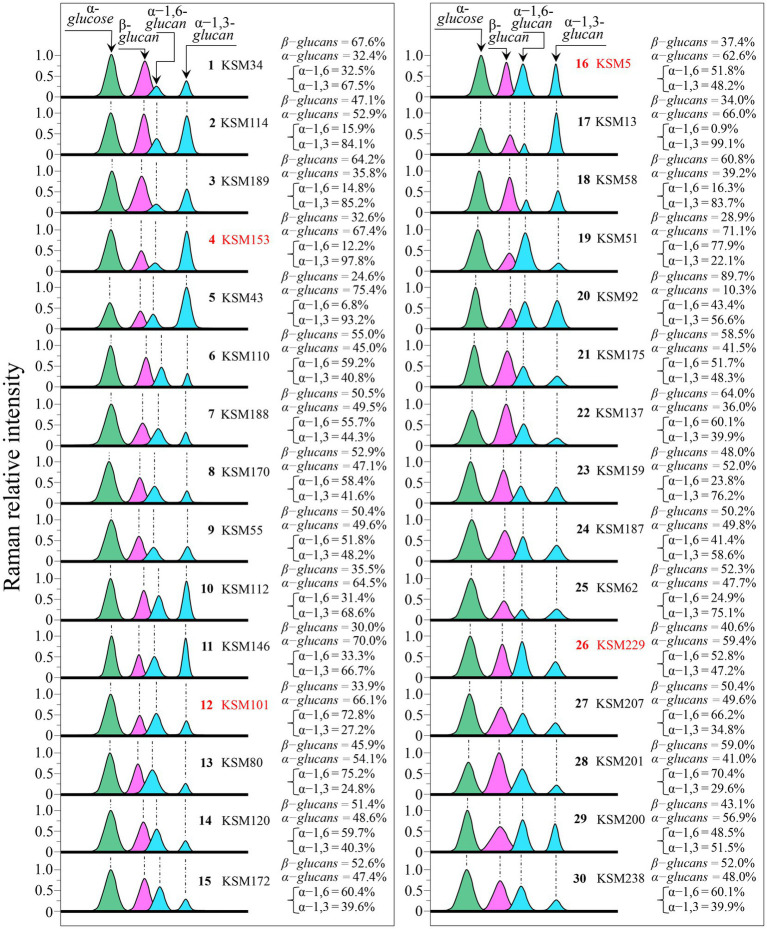
C–O–C stretching signals belonging to β-glucan (at ~890 cm^−1^), α-1,6-linked glucan (at ~919 cm^−1^), and α-1,3-linked glucan (at ~941 cm^−1^) molecules, together with the fingerprint signal at ~851 cm^−1^ from α-glucose rings in free sugar molecules, were extracted from the wavenumber zone D (850–950 cm^−1^) of the high spectrally resolved (average) spectra of 30 bacterial isolates from swab samples after biofilm cleaning and successive culture for 24 h with addition of sucrose (cf. labels in inset corresponding to those assigned in Table 1). The respective fractions of different glucans are listed in inset, as computed according to previous calibrations on elementary compounds (Pezzotti et al., 2023b, 2024). Red labels locate the 4 clinical strains classified as *Cnm*^(+)^*Sm* according to genomic analyses (i.e., samples labeled as 4, 12, 16, and 26 in Table 1).

**Figure 7:**
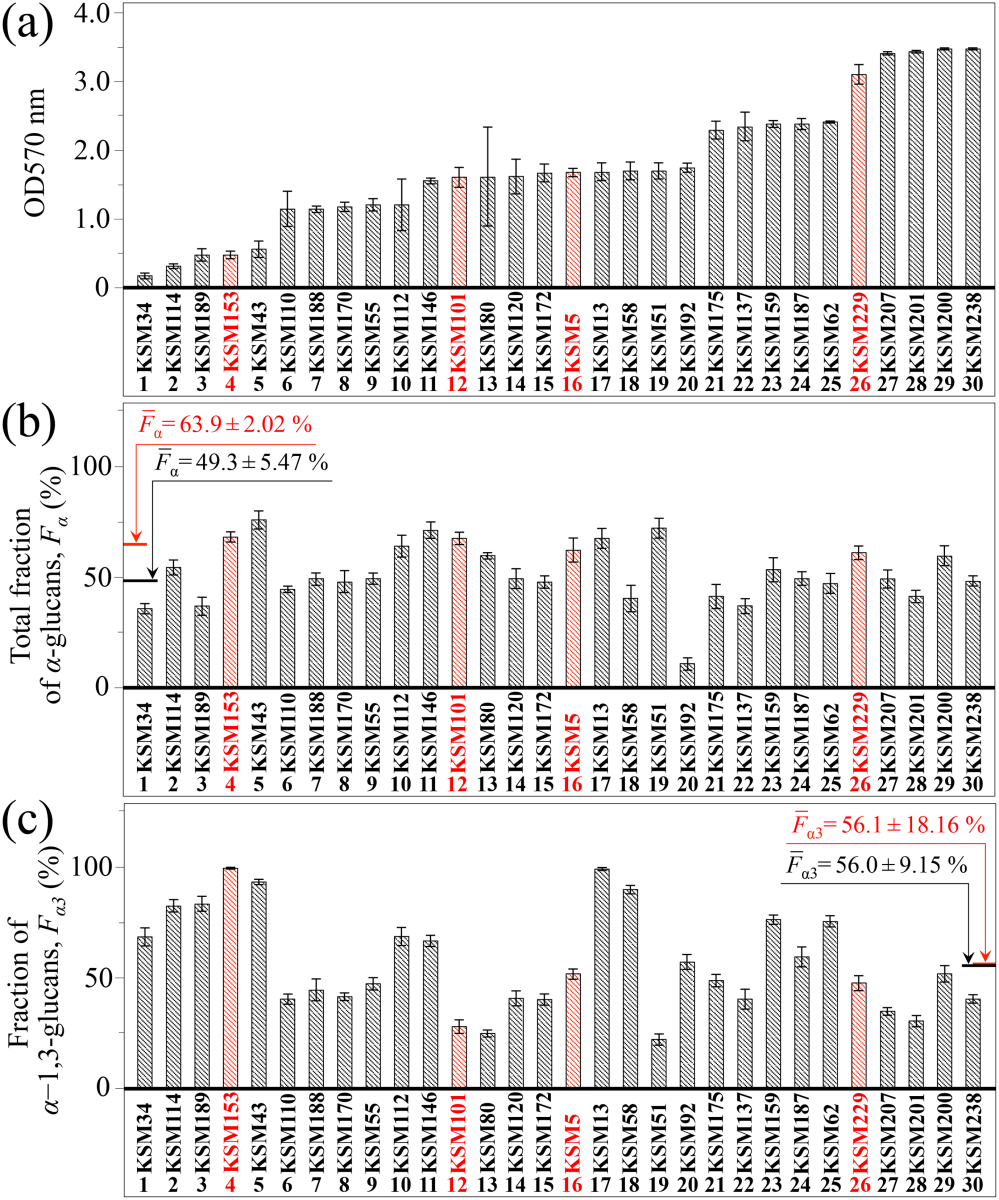
**(a)** Biomass values of isolate biofilms, as determined by crystal violet assay (OD570 nm values), **(b)** total percent fractions of α-glucans, *F*_α_, with respect to β-glucans, and **(c)** percent fractions of α-1,3-linked glucans, *F*_α3_, with respect to the α-1,6-linked ones for the 30 clinical isolates under examination; the comparison failed in locating clear and statistically validated differences between *Cnm*^(+)^*Sm* and *Cnm*^(−)^*Sm* strains (the former ones red-labeled) or among different *S. mutans* sero types (cf. Table 1). Note, however, that the average *F*_α_ value in *Cnm*^(+)^*Sm* strains was ~1.3 times higher than the one in *Cnm*^(−)^*Sm* strains, although the *F*_α3_ values for *Cnm*^(+)^*Sm* and *Cnm*^(−)^*Sm* strains were the same [cf. values in inset to **(b)** and **(c)**].

Raman spectra were collected with high spectral resolution in Zone D, namely, in the spectral interval 850–950 cm^−1^ (cf. Figure 2). In this Raman zone, key signals at ~890, 919, and 941 cm^−1^, which represent spectroscopic fingerprints for β-glucans, α-1,6-and α-1,3-linked glucans, respectively (Wiercigroch et al., 2017), were deconvoluted together with the signal located at ~851 cm^−1^ from α-glucose rings in free sugar molecules. Figure 6 shows these signals as collected on the (average) spectra of the 30 clinical isolates under examination in this study. The respective fractions of different glucans are given in inset, as computed according to previous calibrations (Pezzotti et al., 2023b, 2024). In an attempt to link the biomass of isolate biofilms, as determined by crystal violet assay (cf. Section 2.2), and the above Raman data characterizing biofilm glucan structures, we plotted and compared the OD570 nm value, the total percent fraction of α-glucans, *F*_α_, with respect to β-glucans and the percent fraction of α-1,3-linked glucans, *F*_α3_, with respect to the α-1,6-linked ones. These plots are shown in Figures 7a–c, respectively, for all 30 clinical isolates under examination. However, these plots failed in locating clear and statistically validated differences either between *Cnm*^(+)^*Sm* and *Cnm*^(−)^*Sm* strains or among different *S. mutans* sero types (cf. Table 1). The average value computed for the *F_α_* parameter in *Cnm*^(+)^*Sm* strains was ~1.3 times higher than the one in *Cnm*^(−)^*Sm* strains, while the *F*_α3_ values were the same (cf. values in inset to Figure 7). The relative intensity of the signal located at ~851 cm^−1^ from α-glucose rings in free sugar molecules, which represents the reserve of available energy stored within the biofilm, was always the highest in Zone D, except for two samples (i.e., the samples labeled 5 and 17; cf. Figure 6), which were the richest in α-1,3-linked glucans. These two samples were both *Cnm*^(−)^*Sm* strains and *c* sero type; they presented no other genome-related peculiarities with respect to other isolates. In summary, it was not possible to locate clear structural differences, and thus define appropriate parameters, to differentiate between *Cnm*^(+)^*Sm* and *Cnm*^(−)^*Sm* strains merely from their biofilms structure. This result, which is in line with the preliminary assessment of clinical isolates shown in the companion paper Part II (Pezzotti et al., 2026b), confirms on a larger and statistically significant sampling scale that the biofilm structure of *S. mutans* mostly depends on environmental circumstance and varies with on the pathophysiological state of the bacterium rather than reflecting any specific genomic characteristic of the bacterial strain. In the specific circumstances of the present study, the results also show that the adopted two-days culture set before Raman analyses does not suffice to completely release the stress state of the clinical isolates.

## 4 Discussion

### 4.1 Quantitative spectroscopic parameters for Cnm^(+)^Sm diagnostics

**Figure 8:**
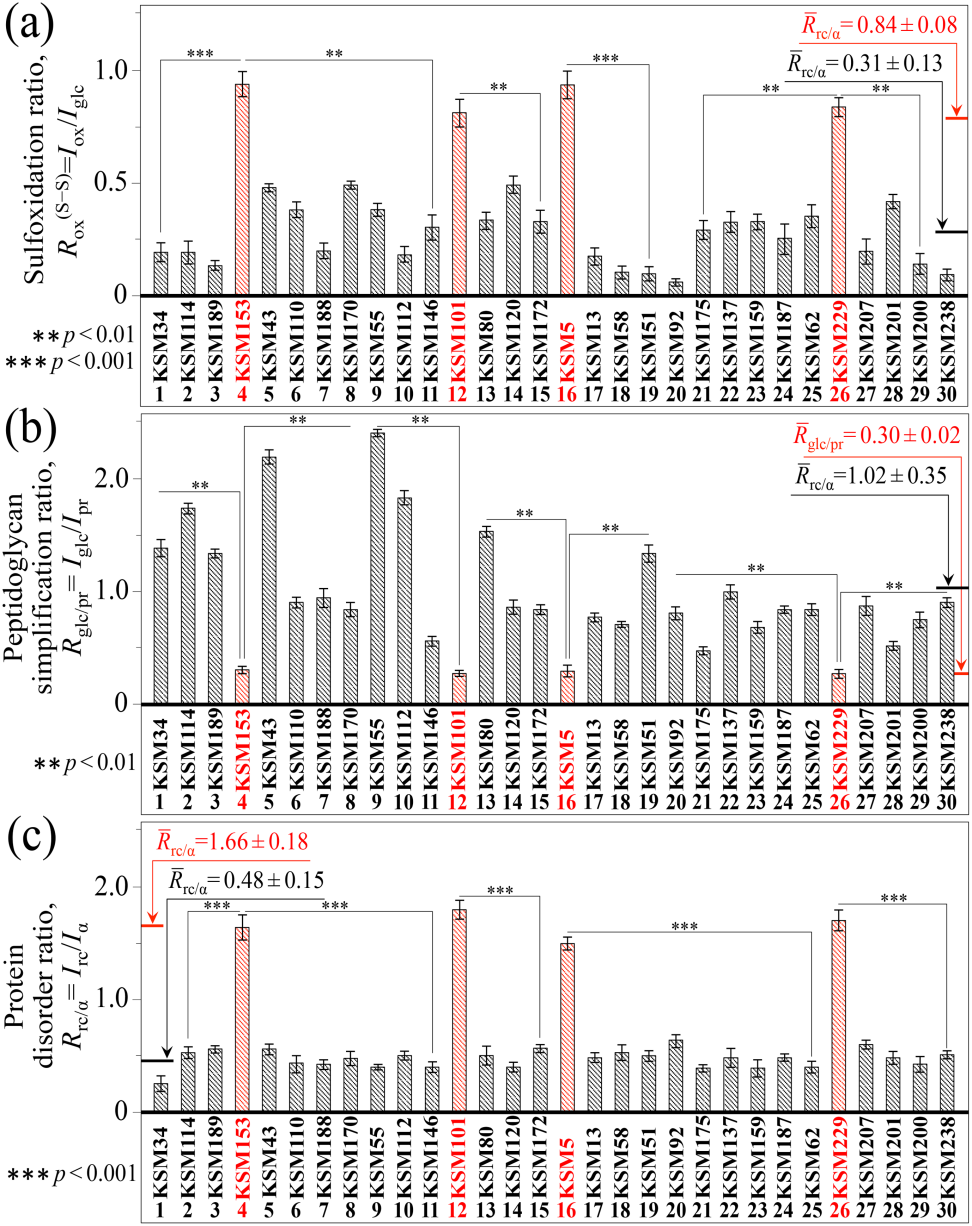
**(a)** Sulfoxidation ratio, *R*_ox_^(S–S)^ = *I*_ox_/*I*_glc_, **(b)** peptidoglycan simplification ratio, *R*_glc/pr_ = *I*_glc_/*I*_pr_, and **(c)** protein disorder ratio, *R*_rc/α_ = *I*_rc_/*I*_α_, as computed for the 30 clinical isolates investigated; the plots show that the 4 *Cnm*^(+)^*Sm* clinical strains (red-labeled; cf. Table 1) can unequivocally located by the proposed Raman spectroscopic procedure with statistical significance (cf. labels in inset) given their clearly higher degree of sulfoxidation, more pronounced membrane peptidoglycan simplification (in favor of the overall protein amount), and higher protein structural disorder (cf. average values and standard deviations in inset).

**Figure 9:**
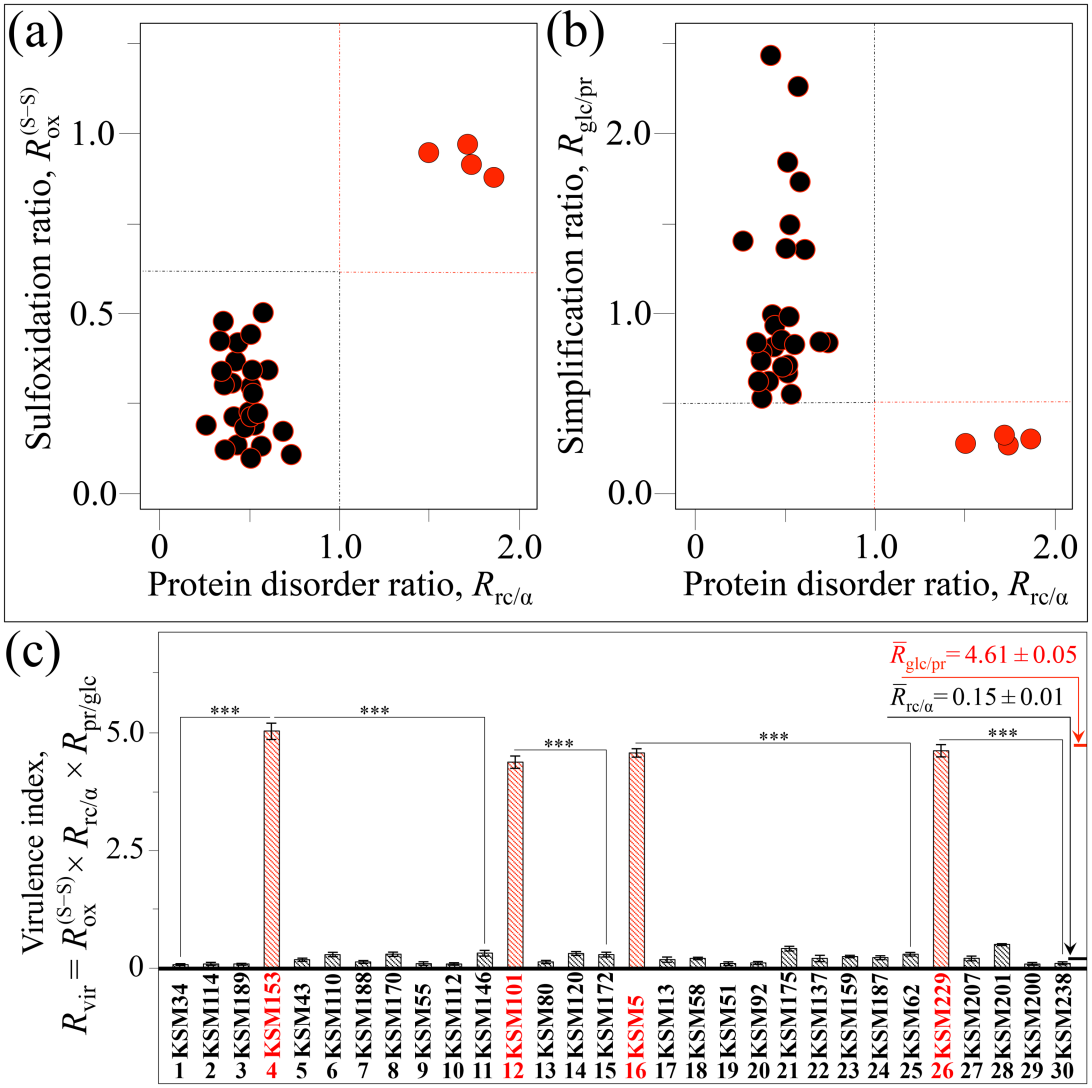
Plots of the *R*_ox_^(S–S)^ **(a)** and *R*_glc/pr_ **(b)** parameters as a function of protein disorder ratio, *R*_rc/α_; both plots could clearly locate *Cnm*^(+)^*Sm* with respect to *Cnm*^(−)^*Sm* ones (red and black data points, respectively), although a more pronounced difference was found in the case of the plot in panel **(a)**. In panel **(c)**, a plot of the virulence index, *R*_vir_, defined as the product of three spectroscopic parameters *R*_ox_^(S–S)^, *R*_rc/α_, and *R*_pr/glc_ (*R*_pr/glc_ = 1/*R*_glc/pr_), is shown for all 30 clinical isolates. This latter parameter singled out the *Cnm*^(+)^*Sm* strains (red-labeled) from the group of 30 with high statistical significance (cf. average values for both *Cnm*^(+)^*Sm* and *Cnm*^(−)^*Sm* strains and respective standard deviations in inset).

In the companion paper Part II (Pezzotti et al., 2026b), spectroscopic classification criteria were proposed for *S. mutans* clinical samples based on Raman algorithms incorporating signals from oxysulfur and peptidoglycans molecules, and Amide I signals from proteins. Three spectroscopic parameters were put forward, as follows: the sulfoxidation ratio, *R*_ox_^(S–S)^, the peptidoglycan simplification ratio, *R*_glc/pr_, and the protein disorder ratio, *R*_rc/*α*_. The parameter *R*_ox_^(S–S)^ is computed from the ratio between the overall area subtended by S–S oxysulfur signals in Zone A (*I*_ox_) and that of the overall peptidoglycan (*GlcN*/*GlcNAc*) skeletal signals in Zone B (*I*_glc_); *R*_glc/pr_ is obtained from the ratio between the above-defined areal intensity *I*_glc_ and the overall spectral area of the protein Amide I signals in Zone C (*I*_pr_); and, *R*_rc/α_ is evaluated from the areal ratio between the random coil (*I*_rc_) and α-helix (*I*_α_) signals in the Amide I Zone C. These spectroscopic parameters are plotted in Figures 8a–c, respectively, for the 30 clinical isolates investigated. These plots show that the 4 *Cnm*^(+)^*Sm* clinical strains (according to genomic analyses) could be spectroscopically located in an unequivocal way and with statistical significance (cf. labels in inset) by virtue of their clearly higher degree of sulfoxidation, more pronounced membrane peptidoglycan simplification (in favor of the overall protein amount), and higher protein structural disorder. Average values for each of the above parameter in *Cnm*^(+)^*Sm* and *Cnm*^(−)^*Sm* strains are given, together with their respective standard deviations, in inset to Figure 8. In Figures 9a,b, plots are shown of the *R*_ox_^(S–S)^ and *R*_glc/pr_ parameters, respectively, as a function of protein disorder ratio, *R*_rc/α_. As seen, in both plots, *Cnm*^(+)^*Sm* and *Cnm*^(−)^*Sm* strains clearly lie in different quadrants, their difference being more pronounced in the case of the plot in (a) involving *R*_ox_^(S–S)^, while the *R*_glc/pr_ parameter shows a higher degree of fluctuation among clinical isolates. Such fluctuations point to a higher dependence of the *R*_glc/pr_ parameter on environmental conditions. In section (c) of the same figure, we defined a virulence index, *R*_vir_, and plotted it for all 30 clinical isolates. *R*_vir_, which is computed as the product of the three spectroscopic parameters *R*_ox_^(S–S)^, *R*_rc/α_, and *R*_pr/glc_ (where *R*_pr/glc_ = 1/*R*_glc/pr_), singled out the *Cnm*^(+)^*Sm* strains from the group of 30 with high statistical significance (cf. labels in inset). Moreover, *R*_vir_ average values for both *Cnm*^(+)^*Sm* and *Cnm*^(−)^*Sm* strains (listed with their respective standard deviations in inset to Figure 9c) differed by a factor 30. The *R*_vir_ parameter can be regarded as the ultimate index of isolate virulence since it condenses the strain capacity to stick on collagen surfaces (through a higher population of sulfoxide compounds at its surface), its survival strategy (less peptidoglycan to gain a Cnm protein coating), and its higher protein structural disorder (favoring flexibility of the bacterial surface, thus facilitating bonding on collagen surfaces). Therefore, this parameter is effective in speciating isolate virulence, because it is comprehensive of the main structural modifications to which *S. mutans* isolates undergo to survive in severe environments.

An additionally important finding was that the *R*_vir_ parameter was unrelated to isolate serotype. The observed structural adaptation, which includes increased expression and surface localization of the collagen-binding Cnm protein, is independent of serotype because serotype classification in *S. mutans* is determined by differences in cell wall rhamnose–glucose polysaccharide side-chain composition rather than by variations in core cell wall architecture or collagen-binding adhesins. The cnm gene is thus not serotype-specific and can be distributed across multiple serotypes through horizontal gene transfer. Therefore, regulation and expression of Cnm, and the associated structural remodeling detected by Raman spectroscopy, are governed by strain-level genetic background and environmental cues rather than by serotype-defining polysaccharide structures. This explains why *R*_vir_ values were observed to vary irrespective of serotype classification.

Another important point resides in the presence of fluctuations in the ratio, *R*_pr/glc_, namely, in the balance between peptidoglycans and proteins in the bacterial structure among different isolates. The observed fluctuations likely reflect strain-specific differences in cell-wall regulatory networks, gene dosage, and environmental responsiveness rather than a uniform structural shift. Expression of the cnm gene is modulated by global transcriptional regulators and stress-response pathways, which vary between isolates due not only to genetic polymorphisms, but also to epigenetic regulation. In parallel, peptidoglycan synthesis and remodeling are dynamic processes influenced by growth phase, metabolic activity, and cell wall turnover rates. Because the Cnm protein is a surface-anchored adhesin integrated into the cell wall matrix rather than a true biochemical substitute for peptidoglycan, its relative abundance will depend on the balance between adhesin expression and basal cell wall biosynthesis. Consequently, inter-isolate variability in regulatory control, metabolic state, and adaptive responses to environmental cues can explain the observed fluctuating *R*_pr/glc_ value across strains.

Finally, the fact that biofilm molecular characteristics were found to be not significantly (or not unequivocally) different in comparing two-days sucrose-cultured *Cnm*^(+)^*Sm* and *Cnm*^(−)^*Sm* strains (cf. Figures 6, 7) show that it is not possible to discern between *Cnm*^(+)^*Sm* and *Cnm*^(−)^*Sm* strains by merely analyzing their biofilms. This circumstance confirmed the parametric study given in the companion Part II (Pezzotti et al., 2026b). While the biofilm results show that *Cnm*^(+)^*Sm* strains did not lose any of the well-known *S. mutans* capacity to synthesize a strong and impermeable biofilm, they also indicate the need to remove the biofilm and directly check the bacterial cell structure in order to locate *Cnm*^(+)^*Sm* strains by Raman analyses.

In summary, the selected Raman parameters well performed as powerful indicators of the structural differences between *Cnm*^(+)^*Sm* and *Cnm*^(−)^*Sm* strains for a large number of clinical isolates, as far as they related to bacterial cell structure (not biofilm structure). Therefore, the present clinical study confirmed that Raman spectroscopy could be at least equivalent to genomic analyses in classifying *S. mutans* bacteria with respect to their Cnm character. Finally, note that the structural characteristics unveiled by Raman spectroscopy did not show any correlation with *S. mutans* serotypes (cf. Table 1).

### 4.2 Hypotheses on molecular origin of oxysulfur compounds in Cnm^(+)^Sm

Sulfur enables microorganisms to exploit various biological functions by virtue of its hypervalence, which permits the occurrence of a number of different oxidation–reduction transformations. Sulfhydryl groups of cysteine residues in proteins have been reported to oxidize and to form disulfide bridges, which play an important role in the stability of proteins by helping in maintaining their tertiary structures (Betz, 1993). In two companion papers Parts I and II (Pezzotti et al., 2026a, b), we showed Raman evidences for enhanced presence of tetrathionate ions (S_4_O_6_^2−^), disulfite ions (S_2_O_5_^2−^), dithionate ions (S_2_O_6_^2−^), dithionite ions (S_2_O_4_^2−^), and thiosulfate ions (S_2_O_3_^2−^) in the molecular structure *Cnm*^(+)^*Sm*. This finding was interpreted as part of a fundamental reshuffling, which included an increased amount of disordered proteins at the expenses of peptidoglycan membrane compounds. As mentioned above, oxidation of sulfur compounds is a strategy commonly followed by microorganisms to adapt their structural components to various purposes; mainly two inorganic forms of reduced sulfur (e.g., sulfide and elemental sulfur) becoming promptly oxidized upon coupling with reduced oxygen or nitrate (NO_3_^−^) (Fry et al., 1988; Burgin and Hamilton, 2008). In the present context, we have suggested that *Cnm*^(+)^*Sm* bacteria exploit oxysulfur compounds to enhance adhesion once accessing the blood circulatory system; the presence of oxysulfur molecules/ions being thus the molecular origin of pathologies related to the formation of microbleeds. The present study tested this hypothesis on a large number of clinical isolates and, in agreement with genomic analyses, confirmed a clear enhancement of oxysulfur molecules only in *Cnm*^(+)^*Sm* strains. Despite this clear result, however, it should be noted that the metabolism of *S. mutans* bacteria is not generally associated with the production of oxysulfur compounds in the way several other microorganisms with specialized sulfur metabolism pathways are (Friedrich, 1997). This leaves open two main issues: (i) how and from which compounds the observed enhancement of the oxysulfur molecules detected in *Cnm*^(+)^*Sm* actually originates; and, (ii) by which mechanism(s) the detected oxysulfur compounds link to collagen and, more specifically, to collagen IV, which is the primary collagen type found in the extracellular basement membrane of endothelial cells (Thomas and Aune, 1978).

**Figure 10:**
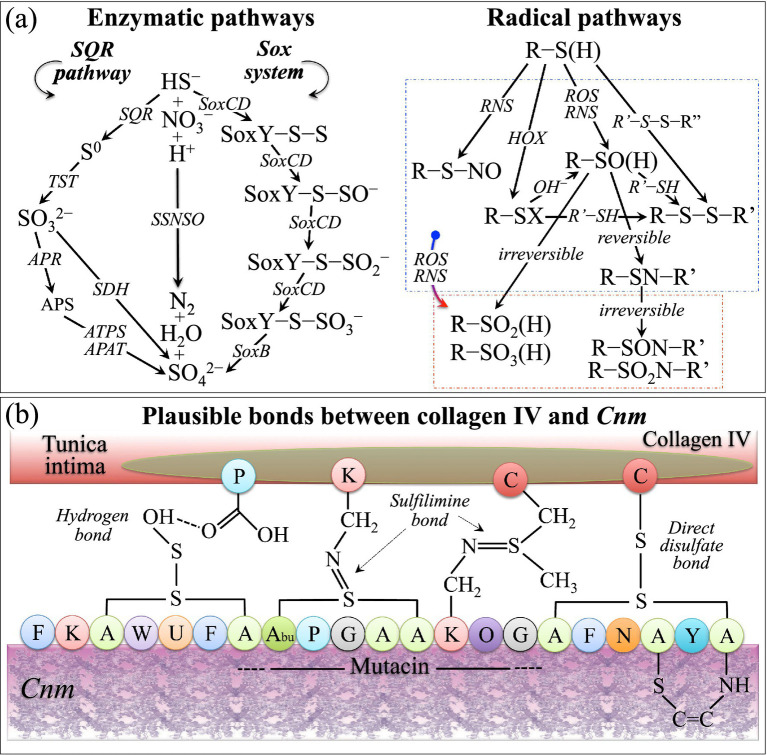
**(a)** Possible enzymatic and radical pathways (left and right sides, respectively), which could enable the formation of oxysulfur molecules in *S. mutans* (replotted and adapted from Refs. Poser et al., (2014) and Lee and Davey (2017); blue and red broken-line squares enclose reversible and irreversible reactions, respectively); and **(b)** plausible types of S–S and S=N bonds linking the *S. mutans Cnm* protein and collagen IV (mutacin structure adapted from Ref. Mota-Meira et al., (1997)).

Regarding the above issue (i), *S. mutans* is known to produce enzymes that involve sulfur-containing compounds and most of its strains are capable to produce mutacins in the oral environment, mainly for self-defense purposes (Pezzotti et al., 2023b; Balakrishnan et al., 2002). Mutacins are a group of lantibiotics consisting of modified peptides including sulfur-containing amino acid residues (i.e., cysteine) involved in post-translational modifications (Merritt and Qi, 2012). An obvious oxidative pathways could thus be figured out with lantibiotic mutacins undergoing oxidative modifications into oxysulfur compounds under conditions of oxidative stress. After forming disulfide bonds, cysteine residues could further oxidize to form sulfones, the formation of sulfoxides and sulfones from cysteine necessarily involving a stepwise oxidation of sulfur from the –SH (thiol) group into a sulfonic acid derivative (Kaiser et al., 2019). On the other hand, oxidation of sulfur could also occur through a variety of enzymatic pathways: (a) sulfur oxidation enzymes (e.g., sulfur dioxygenase), (b) thiosulfate oxidation enzymes (sulfur oxidizing enzyme system, tetrathionate intermediate thiosulfate oxidation pathway, and thiosulfate dehydrogenase), and (c) sulfide and sulfite oxidation enzymes (e.g., sulfide:quinone oxidoreductase) (Poser et al., 2014). Two main enzymatic pathways have so far been suggested for prokaryotic species to oxidize sulfide. One consists in the oxidoreductase reaction with sulfide to donate electrons, which is commonly referred to as the sulfide:quinone oxidoreductase (SQR) pathway (Beller et al., 2006). The SQR pathway starts from the HS^−^ sulfide molecules that produce elemental sulfur, S^0^, which in turn undergoes thiosulfate:cyanide oxidoreductase (TST) to produce sulfite ions, SO_3_^2−^. Sulfite ions can then either feature an adenosine 5′-phosphosulfate reductase (APR) reaction to produce adenosine 5′-phosphosulfate (APS) and catalyze by the coterminous actions of adenosine triphosphate-sulfurylase (ATPS) and APS:phosphate adenylyltransferase (APAT) to yield sulfate, SO_4_^2−^, or directly generate sulfate ions through sulfite dehydrogenase (SDH) enzymatic reactions (Poser et al., 2014). The other reported enzymatic pathway is the sulfur oxidation (Sox) system (Sievert et al., 2008). In Sox system, the cysteine residues of the SoxY protein function as active sites for sulfur oxidation through the sulfur cycle enzyme sulfane dehydrogenase, SoxCD, which catalyzes a six-electron oxidation reaction within the Sox cycle, and the periplasmic thiosulfohydrolase enzyme, SoxB, which catalyzes the final step in the release of sulfate from the SoxY-bond cysteine oxidation cycle (Petri et al., 2001; Quentmeier and Friedrich, 2001; Meyer et al., 2007). In the present context, the hypothesis of the SQR pathway could be supported by published experimental evidences on the highly efficient energy conserving mechanisms involved with sulfur oxidation processes in *S. mutans* (Kelly, 1982, 1989, 1990, 1999), while the high degrees of sensory, regulatory, and metabolic versatility, and the high responsiveness to the environment of *S. mutans* could also justify a possible development of the Sox system (Lemos et al., 2013; Nakanishi et al., 2018). In the case of a restricted availability of molecular oxygen, the alternative electron acceptor nitrate, NO_3_^−^, can also function as a reductant according to a single-step nitrate-dependent sulfide oxidation path (SSNSO) (Rivett et al., 2008). Figure 10a (left side) shows schematic drafts of SQR, Sox, and SSNSO pathways, which could in principle enable the formation of sulfate molecules in *S. mutans*, according to Poser et al. (2014). The final oxysulfur products of thionation reactions, which we directly detected in the present Raman experiments, could thus either represent intermediate products along the Sox system pathway or be the result of further reactions between the final SQR-pathway-formed sulfate molecules and oxygen radicals (Lacombe et al., 1998). Specifically regarding *S. mutans*, Krull et al. (2000) used mass spectrometry and nuclear magnetic resonance to demonstrate that mutacin peptides can undergo substantial oxygenation, this being a clear indication for the accessibility of various sulfur atoms to oxygenation. Notably, however, although the possibility that cysteine residues of extracytoplasmic lantibiotics directly or enzymatically oxidize to form disulfide bonds cannot be discarded (Lee and Davey, 2017), primary candidates in the formation of direct links with collagen IV appear to be the mutacin sulfur bridges between alanine residues; such “external” sulfurs, have been reported as being key factor in the structural stability of *S. mutans*-produced mutacins (Mota-Meira et al., 1997; Smith et al., 2003). In this latter context, thioether bridges have also been located that connected a variety of mutacin residues (Krull et al., 2000). In summary, in a highly efficient metabolic scenario, an altered bacterial metabolism that enhances the production of sulfur compounds and, concurrently, their oxidation could have the twofold effect of further stabilizing extracytoplasmic mutacins while also enhancing their adhesive capacity. Oxidative modifications (such as the formation of oxysulfur molecules) could thus occur either in correspondence of external mutacin sulfur-bridges or directly at cysteine residues, upon exposure to self-produced radicals or during degradation of lantibiotic mutacin in response to an oxidative environment (Premnath et al., 2018). In order to generate oxidized molecules, thiol groups in proteins may undergo one- or two-electron chemistry. During one-electron oxidation, thiol groups, R–SH, convert into thiyl radicals, R–S^•^, which are chemical species participating in free radical chain reactions and, in cascade, become potentially capable of forming any oxidized species generated by two-electron chemistry (Winterbourn and Hampton, 2008). Upon directly reacting with reactive nitrogen species (RNS), e.g., nitric oxide, NO^•^, thiyl radicals can form S-nitrosylated thiol groups, R–S–NO, and then participate in oxygen-dependent pathways producing reactive oxygen species (ROS), e.g., superoxide (Winterbourn and Hampton, 2008). Two-electron processes, which might consist in thiolate reactions with H_2_O_2_, peroxynitrite, and organic hydroperoxides, R–O–OH, form cysteine sulfenic acid as a reactive oxidation product and metastable intermediate to be readily transformed into other oxidative products (e.g., disulfide bonds with cysteines from other proteins, or glutathione and sulfenamides). In addition, sulfenic acids could also operate as intermediates in the formation of highly oxidized and irreversible oxidative/nitrosative products, such as sulfinic and sulfonic acids (R–S(=O)OH and R–S(=O)_2_OH, respectively), sulfinamides (R–S(O)–NR_2_, with R being alkyl or aryl), and sulfonamides (R–S(=O)_2_–NR_2_). Possible radical pathways, which could enable the formation of double or multiple sulfur-bond-containing oxysulfur molecules in *S. mutans* metabolism are schematically summarized on the right side of Figure 10a, according to the cascades of reactions proposed by Klomsiri et al. (2011). The radical pathways shown in Figure 10a describe a reactive cysteine thiol, R–SH, eventually in its thiolate, R–S^−^, or thiyl radical, R–S^•^, forms directly reacting with RNS (e.g., nitric oxide, NO^•^) to form S-nitrosylated thiol groups, R–S–NO. As an additional path, R–SH could also be easily oxidized by ROS, i.e., hydrogen peroxide, organic hydroperoxides, hypohalous acids (HOX), and/or RNS, i.e., peroxynitrite, to form sulfenic acid, R–SO(H). Such a reactive product could in turn be stabilized, react further to form other reversible disulfides, R–S–S–R′ (i.e., possible precursors of the oxysulfur compounds detected by Raman spectroscopy in the present study), and sulfenamides, R–SN–R′, or generate irreversible oxidation products, such as sulfinic acid, R–SO_2_H, sulfonic acid, R–SO_3_H, sulfinamides, R–SON–R′, and sulfonamides, R–SO_2_N–R′.

In order to enable *S. mutans* to produce oxysulfur compounds from lantibiotic mutacins, a series of genomic modifications would be required, facilitating sulfur oxidation, modifying sulfur-containing linkages, and/or newly introducing enzymatic functions capable of directly producing oxysulfur derivatives. In other words, such genomic modifications could either involve changes to existing sulfur-metabolizing pathways or feature the introduction of new genes encoding oxidative enzymes. Mutations in genes encoding enzymes that directly impact on sulfur and oxygen species (e.g., sulfide oxidase and sulfoxide synthase) could alter the normal biochemical pathways; potentially leading to abnormal oxysulfur compound formation (Thomas, 1984; Higuchi et al., 1993). Another pathway to oxysulfur formation could be through enzymes involved in oxidative metabolism of sulfur compounds (e.g., sulfur dioxygenase), which require oxygen radicals to oxidize sulfur (Cheng et al., 2024). Possibly additional mutations leading to increased production of reactive oxygen species (e.g., overexpression of superoxide dismutase and catalase enzymes) could facilitate oxidation in sulfur-containing compounds through the accumulation of oxygen radicals, potentially leading to the formation of sulfoxides or sulfones (Fujishima et al., 2013). In substance, all mutations that affect the activity or regulation of both oxygen- and oxygen-radicals dependent enzymes could ultimately affect the biosynthesis of amino acids or secondary metabolites so that sulfur-containing precursors could undergo oxidation to form oxysulfur compounds.

Regarding the specific mechanism by which oxysulfur compounds link to collagen IV, namely, the above issue (ii), the so far reported main binding mechanism, is a combination of hydrogen bonds and electrostatic interactions between oppositely charged residues, namely, the repeating glycine-proline-hydroxyproline sequence present in the *Cnm A* domains and the triple helices in the collagen IV structure (Esberg et al., 2017; di Cologna et al., 2021). However, the present Raman data newly suggest the possibility of an additional pathway allowing a strong *Cnm*/collagen binding through the exploitation of oxysulfur molecules. Mechanisms of electrostatic attraction could be developed between negatively charged sulfates (or sulfites) at the surface of the *Cnm* protein and positively charged collagen amino acid residues (e.g., lysine) (Christianson and Belting, 2014); hydrogen bonds could also link –OH polar groups in bisulfates or oxygen in sulfates (on *Cnm* side) with polar collagen residues (e.g., hydroxyproline) (Rawal et al., 2021); and, crosslinking with collagen could evolve via reactive sulfenic acids (R–SOH) or sulfinic acids (R–SO_2_H) (Heppner et al., 2018). Note that mutacin sulfur bridges between alanine residues, namely, the “external” sulfurs likely forming a sulfenic acid unit toward the environment, present high reactivity toward thiol groups. Therefore, their primary fate could be the formation of disulfide bonds with cysteine residues from collagen IV. In addition to the above well-established S-related bonding mechanisms, we shall also suggest here the possibility that sulfilimine bonds (N=S) form between collagen IV and the *Cnm* protein (Roy and Gauld, 2024). This hypothesis, which should be tested in future work, is based on two main considerations: (i) the capacity of *Cnm*^(+)^*Sm* to promptly adhere to the endothelial substrate and then strongly resist bacterial translation along the blood flow, which necessarily requires an exceptionally strong chemical bond, such as the N=S one; and, (ii) the fact that N=S bond already occurs at buried locations in collagen IV between methionine sulfur and lysine nitrogen, contributing to the structural stability of the collagen IV network (Vanacore et al., 2009). Although experimental proofs are needed to validate the formation of sulfilimine bonds in correspondence of oxysulfur molecules at the *Cnm*/collagen IV interface, specific hints here suggest that such a strong bond could actually develop. First, the collagen IV structure comprises six highly homologous α-chains, each containing amino-terminal domains rich in both cysteine and lysine residues. Second, following oxidation reactions at –SH thiol groups of *Cnm* protein surface to form sulfenic or sulfinic acids, such highly reactive species could form N=S bonds with nearby nitrogen donors (i.e., lysine residues from collagen IV) (Ronsein et al., 2014). Some of the proposed S-related pathways for chemical bonds between the *Cnm* protein of *S. mutans* and collagen IV on the basement membrane (tunica intima) of endothelial cells are schematically depicted in Figure 10b using, as an example, the *S. mutans* mutacin structure reported by Mota-Meira et al. (1997).

### 4.3 Innovative aspects and limitations of the present Raman findings

One of the major advantages of Raman spectroscopy in oral care is that it can be used as a real-time, multi-tasking, non-invasive, and economically affordable diagnostic tool (Minamikawa, 2022; Qi et al., 2024). The Raman approach does not involve radiations and can be performed on chair-side settings without patient discomfort, thus making it ideal for routine check-ups (Wang et al., 2023). We have shown here that the development of a Raman device fine-tuned for clinical use could be brought into routine oral flora exams, allowing dentists to conduct real-time diagnostic assessments of patients without the need for laboratory-based tests. This accessibility could make it easier (and cheaper) to monitor oral health in a preventive manner, thus counteracting the presently significant economic barrier to develop a large-scale diagnostic program (van der Pol et al., 2021).

The ability of Raman spectroscopy to rapidly and affordably discriminate between *Cnm*^(+)^*Sm* and *Cnm*^(−)^*Sm* isolates directly from human swabs could also have relevant implications for preventive medicine, beyond dentistry-related issues; particularly in the context of predicting the risk of ischemic strokes. Since the enhanced collagen-binding capacity of *Cnm*^(+)^*Sm* isolates (and their peculiar vascular tissue interaction with increased risk of cerebrovascular events), a rapid Raman screening approach could help early identification of individuals whose oral flora is colonized by higher-risk *S. mutans* isolates. In a preventive framework, this technology could thus support risk stratification strategies by identifying patients who may benefit from intensified oral health management, targeted antimicrobial interventions, or closer cardiovascular monitoring. As such, Raman spectroscopy may represent a translational tool linking oral microbial profiling with cerebrovascular risk prevention, potentially contributing to the early mitigation of modifiable risk factors associated with ischemic stroke.

However, despite the presented scientific achievements, the clinical translation of the Raman approach is subject to several boundary conditions. First, the predictive value of identifying *Cnm*^(+)^*Sm* isolates depends on a substantiated and quantitatively defined association between oral colonization and ischemic stroke risk at the population level. At present, large-scale multicenter prospective cohort studies are ongoing in Japan to substantiate this important factor in the frame of the so-called oral-systemic connection (Hosoki et al., 2022). Screening would be most meaningful in individuals with additional vascular risk factors or compromised endothelial integrity, where bacterial–collagen interactions are biologically plausible. At the time of the present Raman study, however, the presence of *Cnm*^(+)^*Sm* strains in the patients’ oral flora should yet be considered a contributory risk marker rather than a standalone causal factor.

Second, for Raman spectroscopy to become widely adopted, several technological achievements need to be met. Main issues reside in the need for dental practitioners to be trained in handling spectroscopic procedures and related algorithms, and in the development of Raman devices that could be integrated into already existing dental practice workflows (Zhang et al., 2022; Rathnayake et al., 2024). Standardized Raman acquisition protocols, spectral preprocessing, and validated classification algorithms should also be built up to ensure reproducibility across operators and clinical settings. In this latter context, a dedicated and easily transportable Raman device should become available at an accessible price. In order to motivate such an effort, more research is required to validate the overall clinical utility of the Raman approach across a wider range of oral health conditions. The present work fully validates a specific *S. mutans*-related procedure and proposes working spectroscopic algorithms for their precise and reliable oral diagnostics. Such procedure, which performs at least as well as genomic analysis, is capable to specify in great detail the structure of *S. mutans* bacteria, their virulence and, ultimately, enables to assess the risk for microbleeds formation their presence is associated with. At the present stage of development, the overall Raman procedure would require at least 24–48 h since the dental practitioner recover a swab sample from the patient; this time being mainly needed for purification and bacterial sample preparation, while the Raman measurement itself only requires few minutes. Several strategies could potentially reduce this preparatory phase. Microfluidic-based bacterial enrichment directly from swab samples could selectively concentrate bacterial cells without extended culture. Filtration and centrifugation protocols combined with short pre-enrichment steps (e.g., 3–6 h incubation) could provide sufficient biomass for Raman detection while maintaining strain representativeness. Finally, coupling Raman analysis with selected Raman tags (e.g., attached to Cnm-protein-specific markers) could allow much faster Raman identification. Once validated, these approaches could enable same-day analysis and enhance the feasibility of Raman-based screening in clinical and preventive settings.

Ongoing research is oriented to minimize the time of sample preparation and to develop an agile Raman instrument more widely dedicated to oral flora analyses. Such efforts will open a new path toward swift and sustainable chair-side intervention, personalized oral care, and ischemic stroke risk assessment.

## 5 Conclusion

Nowadays, the microbiology research community and industry require a simple, fast, and standardized way to obtain phenotypic information and quantitatively measure the virulence of oral bacteria in a way that concurrently takes into account both genomic and metabolomic factors affecting virulence. The Raman methodology discussed in the present three companion papers could ultimately develop into an advanced technology that greatly facilitate and, ultimately, allow standardizing a swift, quantitative, and reliable procedure for assessing virulence in *S. mutans* and related risks of systemic health problems such as heart disease and ischemic strokes. Being capable to comprehensively assess both genomic and epigenetic factors, the presented Raman evaluation of *S. mutans* is not only capable to swiftly distinguish between *Cnm*^(+)^*Sm* and *Cnm*^(−)^*Sm* clinical isolates, but can also serve for clarifying mutations or adaptations on its virulence. Compared to traditional culture/molecular detection, the advantages of Raman technology, including speed, label-free, state sensitivity, and specific application scenarios, such as high-risk caries risk screening, rapid strain virulence typing, has been clearly highlighted. From a more general perspective, the Raman method can be used to classify and benchmark different bacterial strains, while serving as a reliable comparison platform in the elaboration of new drugs. A dedicated Raman device should easily be integrated in high-throughput strategies, thus becoming a key-factor reducing diagnostic costs, as *S. mutans* isolation and genomic screening efforts are both expensive and time consuming. Although further optimizations are yet needed to reduce the time of sample preparation since swab collection from the patient, it is hoped that the presented Raman procedure will open new avenues in early diagnostics and personalized prevention of systemic diseases.

## Data Availability

The original contributions presented in the study are included in the article/supplementary material, further inquiries can be directed to the corresponding author/s.

## Footnotes

3
https://github.com/tseemann/abricate
